# Morphological, Physiological, and Molecular Responses to Heat Stress in Brassicaceae

**DOI:** 10.3390/plants14020152

**Published:** 2025-01-07

**Authors:** Iram Batool, Ahsan Ayyaz, Tongjun Qin, Xiaofen Wu, Weiqi Chen, Fakhir Hannan, Zafar Ullah Zafar, Muhammad Shahbaz Naeem, Muhammad Ahsan Farooq, Weijun Zhou

**Affiliations:** 1 Institute of Crop Science, Ministry of Agriculture and Rural Affairs Key Laboratory of Spectroscopy Sensing, Zhejiang University, Hangzhou 310058, China; iramabbas524@gmail.com (I.B.); ahsanayazmsc2015@yahoo.com (A.A.); 22216020@zju.edu.cn (T.Q.); 22216029@zju.edu.cn (X.W.); 12016012@zju.edu.cn (W.C.); faakhir.mm92@yahoo.com (F.H.); 2 Institute of Botany, Bahauddin Zakariya University, Multan 40162, Pakistan; zafarbzu@yahoo.com; 3 Department of Agronomy, University of Agriculture Faisalabad, Faisalabad 38000, Pakistan; msnaeem@uaf.edu.pk; 4 Zhejiang Provincial Key Laboratory for Water Environment and Marine Biological Resources Protection, College of Life and Environmental Science, Wenzhou University, Wenzhou 325035, China; ahsanfarooq143@yahoo.com

**Keywords:** global warming, thermo-sensing, heat stress, thermo-morphogenesis, heat acclimation, Brassicaceae

## Abstract

Food security is threatened by global warming, which also affects agricultural output. Various components of cells perceive elevated temperatures. Different signaling pathways in plants distinguish between the two types of temperature increases, mild warm temperatures and extremely hot temperatures. Given the rising global temperatures, heat stress has become a major abiotic challenge, affecting the growth and development of various crops and significantly reducing productivity. *Brassica napus*, the second-largest source of vegetable oil worldwide, faces drastic reductions in seed yield and quality under heat stress. This review summarizes recent research on the genetic and physiological impact of heat stress in the Brassicaceae family, as well as in model plants *Arabidopsis* and rice. Several studies show that extreme temperature fluctuations during crucial growth stages negatively affect plants, leading to impaired growth and reduced seed production. The review discusses the mechanisms of heat stress adaptation and the key regulatory genes involved. It also explores the emerging understanding of epigenetic modifications during heat stress. While such studies are limited in *B. napus*, contrasting trends in gene expression have been observed across different species and cultivars, suggesting these genes play a complex role in heat stress tolerance. Key knowledge gaps are identified regarding the impact of heat stress during the growth stages of *B. napus*. In-depth studies of these stages are still needed. The profound understanding of heat stress response mechanisms in tissue-specific models are crucial in advancing our knowledge of thermo-tolerance regulation in *B. napus* and supporting future breeding efforts for heat-tolerant crops.

## 1. Introduction

Agricultural crops are directly affected by environmental temperature. A variety of potential socioeconomic problems have been raised by the frequent occurrence of abnormally high temperatures brought on by global warming, which has led to losses in crop yields and general concerns about agricultural sustainability and food security [1]. Plants cannot move to locations that are more favorable when temperatures rise because they are sessile. In order to address the issues of global food security brought on by climate change, policymakers, farmers, and crop breeders must have a thorough grasp of the molecular underpinnings of how plants adapt and tolerate high temperatures, particularly crops [2].

Heat stress (HS), heat waves, and warming all have diverse effects on plants [3]. Hypocotyl elongation, altered blooming, and other morphological and architectural alterations are all part of thermo-morphogenesis, a developmental phase shift brought on by mild to moderate increases in temperature [4]. Thermo-morphogenesis refers to the influence of temperature on plant morphogenesis, specifically the organization and shape of the plant body. This concept, similar to photo-morphogenesis, which describes the effects of light cues on growth and development, encompasses the effects of temperature cues on plant morphology and development. The core regulators, downstream responders, and up-stream sensors comprise the well-established regulatory network in thermo-morphogenesis [5]. According to the species, the highest limit of the moderate warm temperature group is up to 25 °C for *B. napus* (rapeseed) and up to 27 °C for *Arabidopsis* [6]. HS, as opposed to exposure to high temperatures, continuously hinders plant growth and development and can potentially result in cell damage and death. Agronomic crop production is actually at very high risk due to the possibility of catastrophic losses in agricultural yields caused by large temperature increases [7]. The observed leakage of ions and amino acids can be explained molecularly as a heat-induced biomolecule movement that disrupts the plasma membrane’s homeostasis and significantly changes its permeability and fluidity. Moreover, HS exacerbates energy shortage by decreasing respiratory enzyme activity. Finally, the life cycle is shortened and yields are decreased because HS readily disrupts photosynthetic efficiency.

Reactive oxygen species (ROS) increase significantly when a plant’s optimal temperature rises by 5 °C to 10 °C, leading to permanent oxidative damage, especially in photosystems I and II [8,9]. HS affects plant phenotypes, causing growth retardation and wilting during the vegetative stage, while the reproductive stage sees reduced pollen viability, abnormal fertilization, and poor grain filling [10]. Plants have developed complex mechanisms for heat acclimation, beginning with thermo-sensing, where they detect temperature changes by either sensing intracellular heat or adjusting RNA and protein levels to respond physiologically [5]. Recent studies have thoroughly described thermo-sensors, which are a prominent area of scientific interest. Plants sense high temperatures by a variety of molecular processes, such as chromatin remodeling, RNA structural changes, subcellular translocation, phase separation, and isomerization [11,12]. However, a significant research gap remains in understanding thermo-sensing in HS crops. According to a recent study, TT3.1 transduces temperature information from the plasma membrane to the chloroplast and may be used as a thermo-sensor in high-stress situations [13].

Recent studies indicate that radish has developed a systemic heat shock response (HSR) regulation network [14]. Heat shock factors (HSFs) and heat shock proteins (HSPs) are key activators of HSRs, while heat-responsive signaling molecules (e.g., Ca^2+^, NO, hormones) act upstream of HSFs in *B. juncea* [15,16]. Non-coding RNAs (ncRNAs) and epigenetic modifications also affect HSR gene expression, with unfolded protein responses (UPR) in the endoplasmic reticulum (ER) playing a crucial role. Thermos-sensitive organelles like mitochondria and chloroplasts use various mechanisms to manage high temperatures [17,18]. HEAT-INDUCED TAS1 TARGET2 (*HTT2*) increased the survival rates of *B. rapa* (Chinese cabbage) by promoting thermo-tolerance and decreasing electrical conductivity, extending hypocotyl length, and up-regulating the expression of several heat-shock factor genes (*BrpHsf*), including the *BrpHsfA7a-1*, *BrpHsfB2b-2*, *BrpHsfA7b*, *BrpHsfA2*, and *BrpHsfB2b-1* expression heat-shock treatment under high temperatures stress [19]. Consistent to these findings, Li et al. [20] discovered a regulatory connection between *HTT* and *Hsf* genes in *Arabidopsis* under heat stress. Furthermore, Qian et al. [21] identified a regulatory relationship between *HsfA1a* and *Hsp* genes in *Arabidopsis* treated under heat stress. As molecular processes are unraveled, HSR regulation networks in crop species are being mapped.

Until recently, acquired thermo-tolerance was thought to be a transient phenomenon. However, recent studies have shown that primed plants can maintain thermo-tolerance over an extended period, known as the recovery or memory phase, lasting several days or longer under favorable conditions. During this memory phase, plants retain enhanced stress protection and can survive otherwise lethal recurring HS events. This prolonged thermo-tolerance, termed HS memory or thermo-memory, enables plants to endure daily temperature fluctuations, prolonged HS, and recurring stress. Two key pathways ubiquitin (Ub)-26S proteasome system (UPS) and autophagy mediate intracellular recycling, maintaining cellular homeostasis. The UPS selectively degrades ubi-quitinated proteins but is limited in capacity, unable to process large aggregates, organelles, or protein complexes [22]. To manage these, plant cells rely on autophagy, which plays a critical role in resetting thermo-memory. Research demonstrates that blocking autophagy in *atg* mutants significantly reduces HS tolerance in rapeseed due to the accumulation of insoluble protein aggregates [23]. NEXT TO BRCA1 GENE 1 (*NBR1*), a plant homolog of the mammalian autophagy receptor SQSTM1/p62, mediates the removal of aggregated proteins under HS, preserving proteome integrity [24]. Recent studies reveal *NBR1*’s involvement in degrading the TOC complex during HS, influencing chloroplast protein import and photosynthetic capacity in Brassica species [25]. Other autophagy cargo receptors, such as ATG8-INTERACTING PROTEIN3 (*ATI3*), are also essential for heat tolerance. The disruption of *ATI3* or its interacting partners *UBAC2A* and *UBAC2B* compromises plant thermo-tolerance by impairing selective autophagy in the ER. While *ATI3*-mediated targets remain unknown, these receptors are crucial for ER component recycling during HS in rapeseed [26]. Upon autophagy induction, ATG8 proteins conjugate with phosphatidylethanolamine (PE) to form ATG8–PE, decorating auto-phagosomal membranes, assisting phagophore expansion, and facilitating auto-phagosome–vacuole fusion. Beyond canonical roles, ATG8s exhibit non-canonical functions during recovery from severe HS. ATG8s relocate to swelling Golgi bodies, recruiting CLATHRIN LIGHT CHAIN 2 (CLC2) to aid vesicle budding and Golgi reassembly, bypassing conventional auto-phagosome formation [27]. These findings underscore autophagy’s essential role in proteostasis, thermo-tolerance, and recovery from HS.

This review aims to summarize recent developments in thermo-sensing mechanisms, the differences between thermo-morphogenesis and heat damage, thermal responses, and heat resilience strategies in crops. In this review, we outlined the recent research exploring heat stress responses in Brassicaceae family members as well as in model plants *Arabidopsis* and rice, focusing on the physiological impact with their underlying mechanisms. We further explore new insights into the dynamics of epigenetic change and system biology approaches of this plant family under heat stress. However, due to the scarcity of information specific to Brassicaceae family members, the reviewed findings stem from research on the model organism *Arabidopsis thaliana* and rice.

## 2. Global Warming Poses a Threat to Food Security

**Figure 1:**
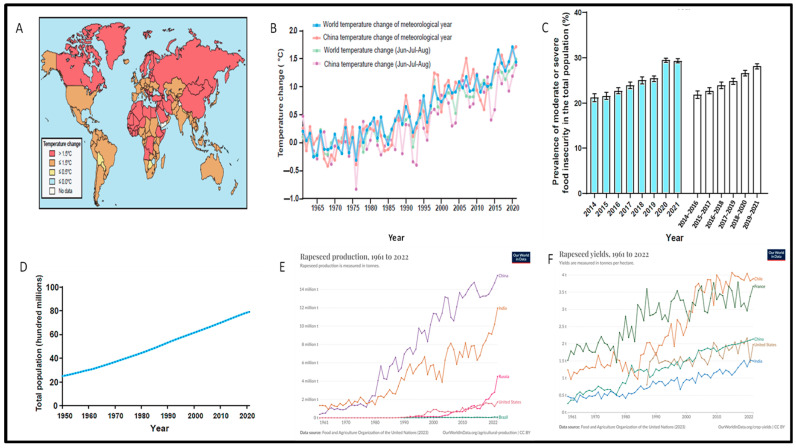
Global warming and rapid population growth pose significant challenges to food security worldwide [5]. (**A**) The mean temperature change across various countries in 2022 is illustrated. (**B**) Additionally, the mean temperature change for meteorological years from 1961 to 2022 is presented. (**C**) The proportion of the population experiencing moderate or severe food insecurity is depicted, with blue columns representing annual values and gray columns indicating 3-year averages. The data are presented as the means with confidence intervals. (**D**) Population dynamics from 1950 to 2021 are also examined. (**E**) A list of the top 10 rapeseed-producing countries based on total production is provided. (**F**) The top 10 rapeseed producers by area harvested are highlighted. All data come from the Food and Agriculture Organization of the United Nations (https://www.fao.org/faostat/en/#data/QCL/visualize (accessed on 22 December 2024)).

Global warming threatens human development and food security. Average global temperatures have increased by 1.25 °C due to human activity, with forecasts suggesting they could exceed 1.5 °C in the next decade [28]. By 2022, several countries, including China, experienced average temperature increases over 1.5 °C (Figure 1A). The impacts of global warming on temperature dynamics are evident worldwide, including China from 1961 to 2022 (Figure 1B). It was reported that crop yields of rapeseed, wheat, rice, maize, and soybean drop by approximately 4.78%, 6.0%, 3.2%, 7.4%, and 3.1% for every 1 °C increase in temperature [29,30]. Improving crop heat resistance is essential, especially given the moderate to severe food insecurity and projected population growth between 2014 and 2022 (Figure 1C,D).

### Heat Stress in Brassicaceae: Effects and Responses

**Figure 2:**
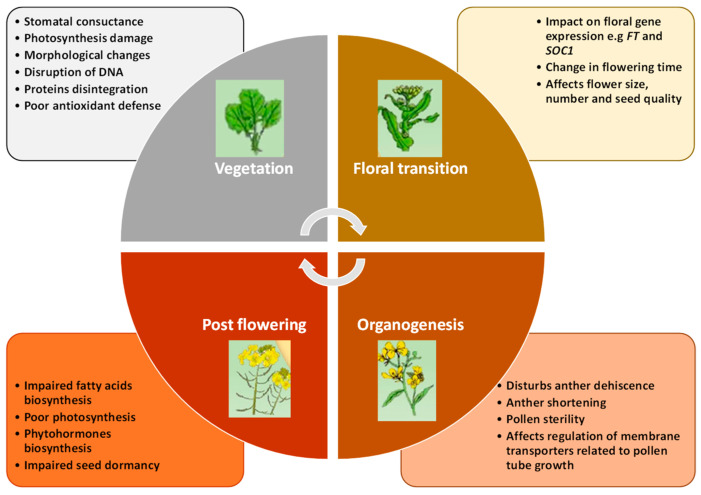
Heat stress-induced changes and physiological response at different developmental stages in *Brassica napus*.

Brassicaceae, one of the largest angiosperm families in the Brassicales order, includes 12–15 tribes, 338–360 genera, and around 3709 species found in tropical and temperate regions [31]. Global warming significantly threatens the production and yield of these species (Figure 1A,E,F). This family comprises many economically valuable species used for food, fodder, medicine, and ornamental purposes [32]. Understanding plant responses to high temperatures and obtaining genetic alleles from germplasm for breeding heat-resistant crops through gene introgression or genome editing is crucial for enhancing global food security in the face of climate change. Thermo-tolerance is regulated both developmentally and tissue-specifically, making it essential to address the effects of high temperatures on different growth stages to develop heat-tolerant cultivars capable of enduring HS (Figure 2).

*Brassica* plants, particularly *B. napus*, are highly sensitive to heat stress, which has become a critical concern due to global climate change (Figure 2). HS significantly affects plant physiology, reproduction, and yield, posing threats to the production and quality of *B. napus* [33]. These physiological disruptions are compounded by the generation of ROS, leading to oxidative stress, membrane lipid peroxidation, and cellular damage. In response, *Brassica* plants activate antioxidant defense mechanisms, including the production of enzymes like superoxide dismutase (SOD) and catalase (CAT), to mitigate ROS damage. During the vegetative stage, HS impairs leaf development, reduces relative water content (RWC), and diminishes plant vigor, leading to stunted growth in *B. napus* [34]. At the reproductive stage, the effects are particularly pronounced, as HS hinders pollen viability, fertilization, and seed development. Male reproductive organs, including anthers and pollen grains, are especially vulnerable, showing abnormalities like pollen sterility, reduced germination, and compromised pollen–pistil interactions. These disruptions translate into fewer flowers, lower seed numbers, and poor seed quality in *B. napus* [33]. Furthermore, high temperatures during seed filling alter oil biosynthesis pathways, reducing the deposition of essential fatty acids and impairing seed storage potential, leading to economic losses in oilseed production. *Brassica* plants exhibit a multifaceted response to HS involving physiological, molecular, and epigenetic adaptations.

Furthermore, HSPs are up-regulated to stabilize denatured proteins and maintain cellular homeostasis. HSFs regulate the expression of HSPs and other stress-responsive genes, ensuring thermo-tolerance via phytohormone signaling like abscisic acid (ABA), salicylic acid (SA), and jasmonates (JA) modulate stress responses by influencing stomatal closure, ROS scavenging, and gene expression [35]. Epigenetic modifications, including DNA methylation and histone acetylation, further fine-tune gene regulation under HS. Studies have shown that polyploidy in *Brassica* species enhances thermo-tolerance by providing genetic redundancy and flexibility, allowing plants to adapt to environmental changes more effectively. Alternative splicing also plays a critical role in stress adaptation by generating diverse protein isoforms that contribute to stress signaling and repair mechanisms in *B. napus* [36]. Transcriptomic and proteomic analyses have identified key genes involved in fatty acid metabolism, stress-signaling pathways, and flowering time regulation, which are crucial for maintaining yield and quality under HS [37]. Notably, genes like *BnWRI1* regulate fatty acid biosynthesis, while flowering integrator genes modulate reproductive timing to overcome unfavorable conditions.

Optimal temperature ranges vary significantly across *Brassica* species [38]. HS occurs when soil and ambient temperatures exceed a plant’s growth and photosynthetic thresholds, causing permanent damage. During early vegetative growth under heat wave conditions, *B. napus* shows increases in saturating light (Asat), transpiration (E), and the intercellular-to-ambient CO₂ ratio (Ci/Ca) under well-watered conditions [39]. These conditions enhance photosynthesis and aboveground growth. However, combined heat and drought stress severely impair photosynthesis, accompanied by a marked reduction in stomatal conductance. Plants respond to overheating by opening their stomata to increase transpiration and prevent leaf damage. During flowering, *B. juncea* exposed to combined heat and drought stress rapidly closes its stomata to reduce water loss, leading to excessive leaf temperature and greater photosynthetic damage [16]. HS also impairs chlorophyll biosynthesis and disrupts photosystem biochemistry, resulting in reduced chlorophyll content, relative water content, and an inefficient antioxidant defense system [36]. Physiological changes during HS, particularly in photosynthesis and transpiration, alter assimilate synthesis and usage, ultimately leading to morphological abnormalities in floral organs in *B. napus* [38]. At the molecular level, HS induces structural damage by disrupting membrane proteins and stability or metabolic damage by impairing enzymatic activities and producing toxic metabolites *B. alboglabra* [27]. Similarly, protein degradation, pigment bleaching, and DNA strand disruption are also consequences of HS in *B. juncea* [16]. Consequently, HS during vegetative and seedling stages initiates a cascade of events affecting reproductive organs later in development, ultimately reducing crop productivity.

To mitigate these challenges, breeding programs are focusing on identifying and incorporating heat-tolerant traits, such as enhanced antioxidant capacity, efficient water use, and robust reproductive development, into *Brassica* cultivars. While considerable progress has been made, there remains a significant knowledge gap in understanding tissue-specific and developmental stage-specific responses to HS. Filling this gap is critical for developing resilient *Brassica* crops capable of withstanding the increasing frequency and intensity of heat waves, ensuring food and nutritional security in a changing climate.

## 3. Thermal Sensing and Responses in Thermo-Morphogenesis

### 3.1. How Plants Sense Warm Temperature

**Figure 3:**
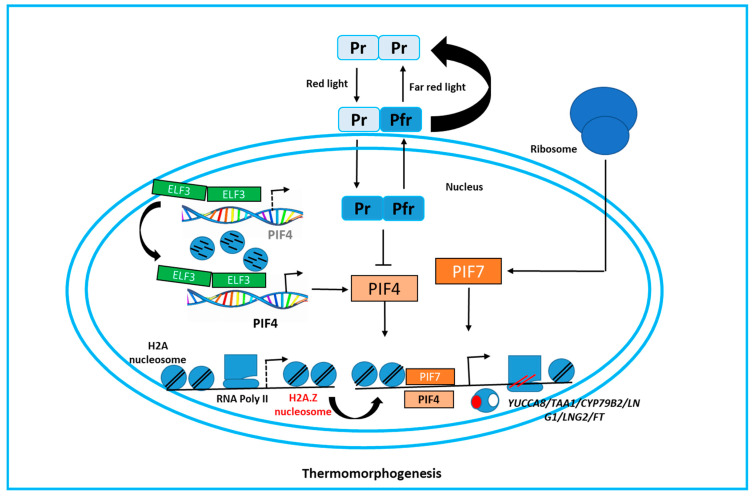
Plants perceive and respond to elevated temperatures through several mechanisms. Increased temperatures activate PIF4 (PHYTOCHROME-INTERACTING FACTOR 4) via the isomerization of phyB and the phase separation of ELF3 (EARLY FLOWERING 3). Additionally, RNA structures act as thermal switches, facilitating the translation of PIF7 (PHYTOCHROME-INTERACTING FACTOR 7). Both PIF4 and PIF7 likely operate upstream of the heat-induced removal of H2A.Z nucleosomes, which ultimately initiates the expression of genes related to thermo-morphogenesis, including YUCCA8 (indole-3-pyruvate monooxygenase 8), TAA1 (L-tryptophan--pyruvate aminotransferase 1), CYP79B2 (cytochrome P450 79B2), LNG1 (longifolia 1), LNG2 (longifolia 2), and FT (flowering locus T protein). Similar heat tolerance mechanism was observed in grain crops [5].

A high temperature term is used to describe a high degree of heat, often to the point of being uncomfortable or burning. Meanwhile, warm temperature implies a mild or more comfortable level of heat that is generally pleasant without burning risk. Recent studies indicate that phytochromes, traditionally known as photoreceptors, also play a role in thermo-sensing, with active isoforms transforming into inactive forms in response to high temperatures [26,27]. Phytochrome B (phyB) is particularly known as a far red-sensitive photoreceptor. Red light converts its inactive form, Pr, into the active homodimer, Pfr [28]. The Pfr homodimer, located in the nucleus, inhibits the basic-helix–loop-helix transcription factors PIF4 and PIF7, which are key regulators of thermo-morphogenesis [29]. High temperatures facilitate the conversion of Pfr back to Pr, relieving the inhibition of PIF4 and PIF7 [30], thereby triggering downstream responses (Figure 3). Emerging evidence suggests that some photoreceptors may also function in thermo-sensing, opening new avenues for identifying thermo-sensors in crops [31]. Additionally, protein phase separation is crucial for thermo-sensing. ELF3 (EARLY FLOWERING 3) inhibits PIF4 mRNA levels through a prion-like domain rich in glutamine. At high temperatures, ELF3 undergoes reversible condensation, forming speckles that sequester ELF3 from the PIF4 promoter [32], subsequently activating PIF4 expression (Figure 3). High temperatures also promote PIF7 translation by causing a hairpin structure to form in the 5′ UTR of PIF7 mRNA [33], influencing its topology based on temperature (Figure 3). This mRNA structure-mediated thermo-sensing is common in plants and warrants further investigation, as similar hairpin sequences have been found in key heat shock regulators like *HSFA2* and temperature-responsive genes such as *WRKY22* [34].

### 3.2. The Molecular Responses of Thermo-Morphogenesis in Plants

The histone H2A.Z is crucial for plants’ detection of environmental temperatures. Nucleosomes with H2A.Z wrap DNA more tightly than canonical H2A, inhibiting transcription by obstructing RNA polymerase II (RNA Pol II) and sequestering transcription factors. Elevated temperatures decrease H2A.Z occupancy, allowing RNA Pol II to access and activate heat-responsive genes like HSF1 [35,36]. Changes in H2A.Z also facilitate PIF4 binding to the FLOWERING LOCUS T promoter, boosting gene expression [37]. Recent studies indicate that PIF-DNA binding may occur before H2A.Z removal under low red/far red (R:FR) light [38]. In low R:FR, PIF7 is dephosphorylated, enabling it to bind target sites and activate downstream genes, potentially through an unknown histone acetyltransferase and the INO80 complex (Figure 3). This suggests chromatin remodelers might not be upstream of temperature sensing, despite responding to low R:FR. Other factors, such as the alternative splicing of FLOWERING LOCUS M mRNA and variations in non-coding RNAs, also affect thermo-morphogenesis in Chinese cabbage [39,40]. At higher temperatures, PIF4 enhances transcription of growth-promoting genes like FLOWERING LOCUS T and CYP79B2, while repressing SPCH, vital for stomatal formation (Figure 3). Additionally, the kinase MAP4K4, encoded by TOT3, plays a role in thermo-morphogenesis in plants [41,42,43]. PIF7 translation is initiated at higher temperatures when a hairpin structure forms in its mRNA’s 5′ UTR (Figure 3).

HS significantly affects protein homeostasis in plants, causing the misfolding of newly synthesized proteins and the denaturation of existing ones. HSPs, including ATP-independent small HSPs (sHSPs) and ATP-dependent HSPs like HSP70 and HSP90, are critical in preventing protein aggregation and maintaining functional conformations. Moreover, sHSPs capture misfolded proteins under stress and transfer them to HSP70s for renaturation, playing a vital role in both basal and acquired thermo-tolerance in Chinese cabbage [44]. HSPs are also linked to DNA repair pathways, further emphasizing their importance in stress responses. HSFs regulate HSP expression and are key in HS response. HSFA1, a master regulator, triggers the expression of downstream HSFs and other transcription factors, including HSFA2, HSFA3, and DREB2A, which coordinate thermo-tolerance mechanisms. HSFA2 prolongs thermo-tolerance by sustaining HSP expression, while DREB2A interacts with NF-Y complexes to regulate HSFA3 under HS in *B. oleracea* [45]. Additional HSFs, such as HSFA4a and HSFA8, act as ROS sensors, linking oxidative stress to heat responses. Transcriptomic and metabolomic analyses of heat-tolerant (‘WYM’) and heat-sensitive (‘AJH’) Chinese cabbage identified 656 differentially expressed genes (DEGs) and 1973 splicing events under high temperature [46]. GO and KEGG analyses revealed enrichment in protein folding, photosynthesis-antenna proteins, and spliceosome pathways. Altered expression levels of 454 transcription factors across 49 families highlighted their role in heat tolerance in *B. rapa* [47]. In *B. rapa*, HSP101, HSP70, and sHSPs were identified as hub genes, with HSP17.6 and HSP70-8 being linked to protein folding and aggregation prevention [48]. The integration of transcriptomic and functional studies provides insights into the regulatory networks and hub genes underpinning heat tolerance in *B. rapa* [47]. These findings support the hypothesis that HSPs and the associated chaperones are critical for mitigating heat-induced damage and enhancing thermo-tolerance in plants.

### 3.3. Putative Thermo-Sensing in Plants

HS thermo-sensing is not as well understood as that of warm temperature sensing. Through chemical interactions, messenger molecule activation, or structural alterations, proteins referred to as thermo-sensors can detect high temperatures in crop plants [44]. We present three possible thermo-sensors that respond to HS in plants.

#### Heat-Induced Cell Wall Remodeling Releases Ca^2+^

**Figure 4:**
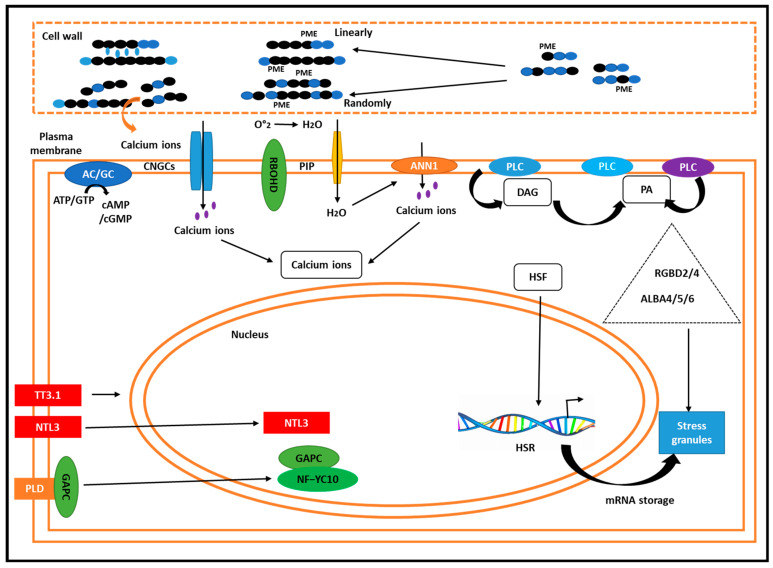
Putative thermo-sensing mechanisms in plants during HS involve several intricate processes. Temperature elevations induce cell wall remodeling through PMEs, leading to a Ca^2^⁺ influx from the apoplast into the cytoplasm. The plasma membrane demonstrates thermo-sensitivity in its fluidity, activating various second messengers including Ca^2^⁺, cAMP/cGMP, ROS, and signaling lipids to relay temperature signals and elicit downstream physiological responses. Ca^2^⁺ enters the cell through Ca^2^⁺-permeable channels and associated proteins, such as cyclic nucleotide-gated channels (CNGCs) and annexins (ANNs). CNGCs are activated by cAMP/cGMP, while ANNs are stimulated by H₂O₂, a significant ROS. Interestingly, Ca^2^⁺ also plays a role in generating ROS. Phospholipid PAs, produced by phospholipase D (PLD) and phospholipase C/DAG kinase (DGK), serve as signaling molecules that target downstream proteins. Inositol phosphates, generated by phospholipases, trigger Ca^2^⁺ release. Proteins RGBD2/4 and ALBA4/5/6 detect HS and promote the formation of stress granules (SGs) through phase separation, thereby protecting heat shock response-related mRNA. The heat-dependent nuclear translocation of glyceraldehyde-3-phosphate dehydrogenase (GAPC) and NTL3 further activates the expression of downstream genes. Additionally, TT3.1, which relocates from the plasma membrane to the endosome in response to HS, functions as a potential thermo-sensor. Similar heat tolerance mechanism was observed in grain crops [5].

The cell wall is the first line of defense in the plant cells because it is heated before the plasma membrane. De-esterified pectin residues are stored with apoplastic Ca^2+^ under normal conditions. Pectin methylesterases (PME) are randomly activated on the pectin by mild HS, which causes the pH of the cell wall to decrease. Protons are released due to dimethyl esterification. The activation of endo-poly galacturonase leads to the release of Ca^2+^ from apoplasts and the weakening of the cell walls (Figure 4). The HSR is finally activated by Ca^2+^ entering the cytoplasm through putative Ca^2+^-permeable channels. High ambient temperatures affect flowering time through H2A.Z-mediated mechanisms in *B. napus* [45].

### 3.4. Activation of Chemical Messengers in the Plasma Membrane

Proteins and lipids are the most thermo-sensitive components in cells [46]. High temperatures alter lipid composition, disrupting fatty acid structures and affecting bilayer rotation, diffusion, and packing density in plasma membranes. These changes in membrane fluidity can affect protein activity, motility, and folding [47]. Given its temperature sensitivity, the plasma membrane acts as a thermo-sensing system. Biological membranes, composed of ordered lipids and proteins, are particularly vulnerable to temperature fluctuations. High temperatures can disrupt lipid structure, affecting head group packing, rotation, and diffusion, which in turn influence membrane protein dynamics [48,49]. The plasma membrane’s sensitivity allows it to function as a thermo-sensor [50,51]. Certain membrane proteins can convert signals from apoplastic chemical messengers into increased cytoplasmic Ca^2+^ ion levels, suggesting they play a role as thermo-sensors in plants.

In Chinese cabbage, like rapeseed, cyclic nucleotide-gated channels (CNGCs) facilitate heat-triggered Ca^2+^ influx [52,53]. CNGCs respond to heat shock similarly to osmo-sensors like hyper-osmolality-gated Ca^2+^ channels (Figure 4). Additionally, adenylyl/guanylate cyclase produces cyclic AMP (cAMP) and cyclic guanosine monophosphate (cGMP), which may act upstream of CNGCs, potentially functioning as thermo-sensors since cAMP/cGMP can activate these channels (Figure 4). Research indicates that ANN1 is activated by H_2_O_2_ and inhibited by MYB30 at the mRNA level, raising questions about whether H_2_O_2_ or Ca^2+^ acts upstream [53]. It has been shown that heat-induced increases in Ca^2+^ require ANN1 (Figure 4). A well-characterized dual Ca^2+^/ROS pathway in salt stress responses illustrates how Ca^2+^ channels and respiratory burst oxidase homolog D (RBOHD) control this mechanism. This process generates self-propagating Ca^2+^/ROS waves through the interaction of Ca^2+^ and ROS. ROS accumulates due to the activation of *RBOHD* in response to HS. The downstream function of *ANNs* is to detect ROS and facilitate Ca^2+^ flow. Recent studies indicate that elevated cytoplasmic Ca^2+^ levels activate *RBOHD* [54], potentially enabling plants to sense temperature (Figure 4). Alongside Ca^2+^ and ROS, lipids also act as signaling molecules that convert external inputs into intracellular responses [55,56]. Phospholipase D (PLD) and phospholipase C/diacylglycerol [DAG] kinase (PLC/DGK) generate phosphatidic acids (PAs), which display dynamic variations and respond rapidly to HS. Due to the elevated concentration of negatively charged PA, the plasma membrane becomes unstable, affecting the activities of membrane proteins. The production of lipids and temperature perception are closely linked, as PAs can bind directly to downstream proteins, influencing various processes. While phospholipase D (PLD) plays a crucial role in heat-induced PA responses, it acts after the primary thermo-sensing pathway since it requires Ca^2+^ and H_2_O_2_ for activation. Phospholipase C (PLC) hydrolyzes phosphatidylinositol 4-phosphate and phosphatidylinositol 4, 5-bisphosphate, producing inositol-1, 4, 5-trisphosphate and diacylglycerol (DAG).

### 3.5. Phase Separation at Protein Levels

The widespread reduction in translation initiation results in the formation of stress granules (SGs), which rely on the liquid–liquid phase separation (LLPS) of proteins. For RNA-binding glycine-rich D2 (RBGD2) and RBGD4 to demonstrate thermo-tolerance, they must condense into heat-induced SGs via LLPS [57]. Once inside these SGs, RBGD2 and RBGD4 can interact with heat-responsive mRNA and other SG proteins, activating further heat responses (Figure 4). Additionally, LLPS under HS is facilitated by acetylation, which reduces the binding affinity of proteins, allowing them to form SGs and processing bodies. This process also stabilizes HSF mRNAs, enhancing thermo-tolerance (Figure 4). These findings underscore the crucial role of protein structure in sensing high temperatures.

### 3.6. Subcellular Location-Specific Changes at Protein Levels

Heat shock-induced subcellular translocation, which alters how proteins interact with other molecules, is another defensive mechanism against thermo-perception in plants. It was reported that migration toward the nucleus is a typical trend. For instance, glyceraldehyde-3-phosphate dehydrogenase, a cytoplasmic glycolytic enzyme, migrates to the nucleus in response to high temperatures, where it influences and activates the transcription factor nuclear factor Y subunit C10 (NF-YC10) [58], allowing signals to be transmitted (Figure 4). At high temperatures, the transcription factor NTL3, which is involved in NAM, ATAF1/2, and CUC1/2 [NAC], moves from the membrane to the nucleus, causing downstream HS reactions (Figure 4). Nuclear translocation and intercellular organelle communication are two of the ways in which plants sense temperature increases [59,60]. To respond to and decipher extracellular HS signals, TT3.1, which is located at the plasma membrane, can enter intracellular endosomes (Figure 4). These results suggest that a key component of thermo-sensing is protein translocation.

## 4. Thermal Tolerance Response in Plants

### 4.1. Transcriptional Regulation: The Central Roles of HSF and HSP Genes

**Figure 5:**
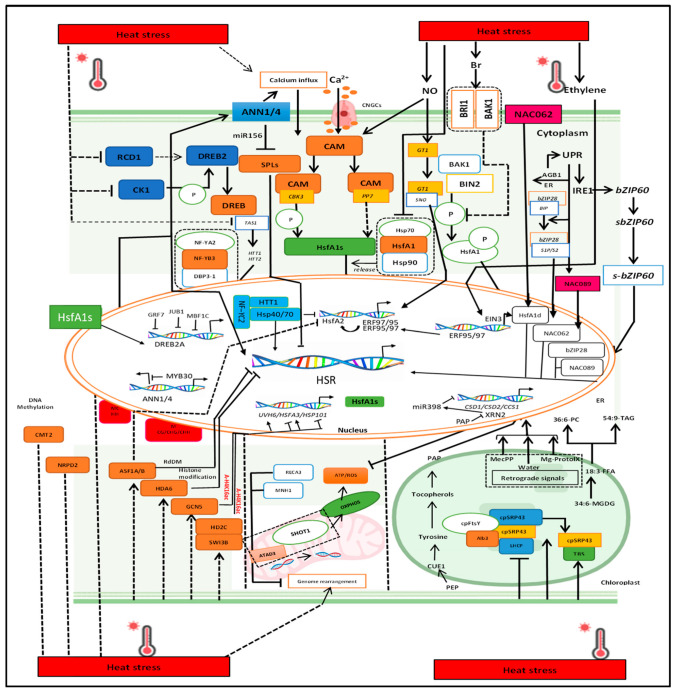
Molecular responses to HS involve a complex network of HSFs and HSPs that mediate HSRs. HS activates HsfA1s by relieving inhibition from Hsp70 and Hsp90, subsequently activating HsfA2 and DREB2A, which regulate HSR gene expression. Under normal conditions, DREB2A is tightly regulated, but during HS, its expression is enhanced by HsfA1s, JUB1, and MBF1c in a trimer-dependent manner. Signaling molecules like PP7 and CBK3 modulate HsfA1 activity, while ethylene and GT-1 promote HSR gene expression through specific transcriptional cascades. Additionally, heat-induced microRNAs (miR156 and miR398) and siRNAs are involved in regulating HSR inhibitors and antioxidant enzymes. The UPR is triggered in the ER, leading to the splicing of bZIP60 mRNA. Various proteins and pathways, including NAC transcription factors and mitochondrial regulatory factors, further influence HS responses. Chloroplast signaling also contributes to thermal response mechanisms, with retrograde signals and lipid metabolism adjustments playing key roles in thermo-tolerance. Overall, these interconnected pathways ensure the effective plant adaptation to HS. Similar heat tolerance mechanism was observed in Arabidopsis [5].

HSR gene expression is primarily responsible for heat responses; it also provides plants with heat shock adaptations and plays several roles in secondary pathways. According to reports, members of the HSF family, known as HsfA1 proteins, serve as master activators of HSR genes, which help in regulating the thermo-tolerance of Chinese cabbage, *Arabidopsis*, and *B. rapa* [61,62,63]. Temperature affects protein activity and gene expression levels. At room temperature, HSP70, HSP90, and HsfA1s combine to generate a compound that inhibits HsfA1s activity and keeps them out of the nucleus, which stops HsfA1 proteins from being activated in Chinese jujube [64,65]. The complex dissociates due to HS activating inactive HsfA1s (Figure 5). For example, SUMOylation and phosphorylation can positively or negatively affect HsfA1 activity. HsfA1 protein activity may also be impacted by post-translational modifications (PTM) [66]. To encourage downstream transcription, for example, phosphorylated HSF1 can use LLPS to create tiny nuclear condensates that can be distributed. The dispersal of HSF1 condensates stops HS responses, making this process reversible in crop plants [67]. DREB2A (DEHYDRATION-RESPONSIVE ELEMENT BINDING 2A) is a key downstream protein of HsfA1, regulating heat and drought responses while managing protein and mRNA levels during heat shock. GRF7 (GROWTH-REGULATING FACTOR 7) typically suppresses DREB2A, but during HS, the destabilizer RCD1 (RADICAL-INDUCED CELL DEATH 1) is degraded. This allows HsfA1s, JUB1 (JUNGBRUNNEN 1), and MBF1c (MULTIPROTEIN BRIDGING FACTOR 1C) to enhance DREB2A mRNA levels. CK1 (CASEIN KINASE 1) phosphorylates DREB2A’s negative regulatory domain (NRD) [68,69,70], controlling its degradation; HS decreases NRD phosphorylation, increasing DREB2A levels (Figure 5).

### 4.2. Role of Signaling Molecules Including Ca^2+^, ROS, and Nitric Oxide

Calmodulin (CaM) decodes Ca^2+^, functioning as a secondary messenger during HS by binding to CaM. Calmodulin-binding protein kinase 3 (CBK3) phosphorylates HsfA1 proteins [71], influencing their ability to bind to downstream target promoters in *B. oleracea* (Figure 5). It is unclear whether PP7, a CaM-binding phosphatase that interacts with HsfA1s, directly dephosphorylates them (Figure 5). Additionally, ROS, such as superoxide [O2^°−^], hydrogen peroxide [H_2_O_2_], singlet oxygen [1O_2_], and hydroxyl radicals (°OH), act as metabolic or signaling molecules in *Arabidopsis* [72,73,74]. These ROS are produced by intracellular organelles like mitochondria, peroxisomes, and chloroplasts, or are generated by nicotine amide adenine dinucleotide phosphate.

### 4.3. ROS-Mediated Phytohormes Signaling in the Plant Cell

The accumulation of ROS damages cells, prompting antioxidant and ROS-scavenging pathways to maintain redox balance and HS adaptation [75]. Extracellular hydrogen peroxide (H_2_O_2_) acts as a signaling molecule that activates plant sensors, such as the leucine-rich-repeat receptor kinase HPCA1, which detects H_2_O_2_ by analyzing cysteine residues and subsequently increases Ca^2+^ levels [76]. Whether these sensors contribute to HS responses remains unclear. Additionally, H_2_O_2_ may be present before HsfA1 activation, initiating the transcription of heat shock-related genes. Similarly, reactive nitrogen species also enhance the binding of transcription activators to the *HsfA2* promoter during HS in *B. alboglabra* by S-nitrosylating the transcription activator GT-1 through S-nitrosoglutathione [27]. Plant hormones play various roles in cellular responses to HS; for example, ethylene activates transcription factors ERF95 and ERF97 via the essential EIN3, increasing *HsfA2* expression and establishing baseline thermo-tolerance [77]. Brassinosteroids (BRs) also positively regulate thermo-tolerance in *B. rapa* and *Arabidopsis*. Without BRs, *HsfA1d* interacts with and is phosphorylated by BIN2, reducing its nuclear localization and DNA-binding affinity [46,78,79]. However, BRs significantly inhibit BIN2 activity, allowing HsfA1d to maintain nuclear translocation and DNA-binding capability [79], ultimately triggering heat shock responses (Figure 5).

### 4.4. Role of ncRNAs in Plant Response Under Heat Stress

Plant non-coding RNAs (ncRNAs) play a crucial role in the epigenetic regulation of HSRs by acting upstream of key genes and transcription factors. MicroRNAs (miRNAs), a type of ncRNA, target messenger RNAs (mRNAs) to either enhance or inhibit translation [80,81,82]. Under normal conditions, SPL (SQUAMOSA PROMOTER BINDING PROTEIN-LIKE) transcription factors regulate HSR genes. However, during HS, miR156 isoforms significantly increase, post-transcriptionally suppressing SPL gene expression and triggering HSR gene activation [83,84,85], which enhances HS memory (Figure 5). Recent advances in large-scale transcriptome sequencing have uncovered a wide array of long non-coding RNAs (lncRNAs) transcribed from eukaryotic genomes [86]. These PROMPTs can extend several kilobases upstream of the transcription start sites of protein-coding genes. Despite the growing catalog of lncRNAs, most remain poorly characterized, with major challenges including the identification of their regulatory targets and understanding their biological roles. Evidence increasingly suggests that lncRNAs regulate transcription by influencing chromatin remodeling [87,88]. Depending on the factors they interact with, lncRNAs may act as transcriptional activators or repressors. Some operate locally at their synthesis sites, modulating nearby gene expression, while others function as trans-acting factors, targeting distant loci. However, the mechanisms by which specific lncRNAs target distinct DNA regions and produce differential effects remain elusive. A notable aspect of the HSR is the heat-induced expression of lncRNAs [89]. Despite minimal sequence homology, these lncRNAs act in trans as direct repressors of Pol II activity, inhibiting basal transcription during HS [90]. However, how the up-regulation of stress genes aligns with the down-regulation of housekeeping genes during HSR remains unresolved.

### 4.5. Epigenetic Modification in Response to Heat Stress

The heat response in plants is intricately controlled by various epigenetic mechanisms, including chromatin remodeling, DNA methylation, and histone modifications. In *Arabidopsis*, thermo-tolerance is inversely related to the DNA methylation levels associated with CMT2 (chromomethylase 2) and CHH (where C is cytosine and H is any base except guanine) in crop plants [86,87]. According to reports, NRPD2, a key component of RNA Polymerase IV and V, is a positive regulator of thermo-tolerance. Additionally, RNA-directed DNA methylation (RdDM) of remnant transposable elements is known to activate nearby protein-coding genes [88,89], underscoring the importance of DNA methylation in thermo-tolerance regulation (Figure 5).

Epigenetic regulators, including acetyl-transferases, methyl-transferases, deacetylases, and demethylases, modify histones to control gene expression during HS [90,91]. The histone acetyltransferase GCN5 promotes thermo-tolerance by enhancing H3K9/K14 acetylation in the promoters of HSFA3 and UVH6 in plants [92]. In contrast, the de-acetylation of H4K16 by the transcriptional repressor HD2C under HS interacts with the SWI3B subunit of the SWI/SNF chromatin-remodeling complex [54,92], leading to the reduced expression of HsfA3, Hsp101, and other HSR genes (Figure 5). Histone deacetylase 6 (HDA6) positively regulates thermo-tolerance and is linked to gene expression reduction via RdDM (Figure 5). Nucleoporin HOS1 and the phosphatase PP2AB0 b-dependent HDA9 relocate from the cytosol to the nucleus in response to heat shock [93,94], where they enhance heat shock signaling (Figure 5).

### 4.6. Molecular Regulation of Heat Stress in Semiautonomous Organelles

HS induces chromosomal rearrangements in semiautonomous organelles like mitochondria and chloroplasts. This process is inhibited by proteins such as RECA3 and MutS homolog 1 (MSH1), with plants exhibiting enhanced thermo-tolerance and significant mitochondrial genome rearrangement in the *msh1* and *reca3* double mutant [33]. Complexes IV and I facilitate oxidative phosphorylation (OXPHOS), a vital process for generating ROS and ATP in mitochondria [95,96]. The protein SHOT1, which interacts with mitochondrial transcription termination factors and ATAD3 (ATPase family AAA domain-containing protein 3) [97], helps maintain the mitochondrial nucleoid structure. Chloroplasts are also susceptible to HS, disrupting photosynthesis and gene expression. The import of nuclear-encoded proteins into chloroplasts is crucial for plant HS tolerance. Chloroplast pre-proteins are directly SUMOylated by the SUMO ligase *AtSIZ1*, and phosphorylation may follow SUMO3 conjugations (Figure 5). It was reported that these mechanisms contribute to thermo-tolerance in *Arabidopsis* by ensuring appropriate protein import into chloroplasts [98]. In chloroplasts, HIL1 (heat-inducible lipase 1) converts 34:6-monogalactosyl di-acylglycerols into 18:3-free fatty acids, leading to changes in lipid composition during HS [99,100]. These fatty acids are subsequently transformed in the ER into 36:6-phosphatidylcholines and 54:9-triacylglycerols (Figure 5). Additionally, the molecular chaperone cpSRP43 protects tetra-pyrrole biosynthesis (TBS) by stabilizing light-harvesting chlorophyll-binding proteins in the thylakoid membrane, preventing aggregation during HS [101,102,103]. During HS, cpSRP43 interacts with cpSRP54 to facilitate the integration of light-harvesting chlorophyll a/b proteins into the thylakoid membrane [104,105]. The stress response enables free cpSRP43 to attach to TBS proteins, further preventing aggregation (Figure 5).

The thylakoid membrane Ca^2+^ sensor elevates stromal Ca^2+^ levels, activating transcription factors and CaM/Ca^2+^-binding protein kinases that initiate signaling pathways in the nucleus. Mg-protoporphyrin IX, a conventional tetrapyrrole, binds to HSP90 [106], facilitating the accumulation of sHSPs through the interaction of HSP90.1 and the peptidyl prolyl isomerase ROF1 (rotamase FKBP1) [107]. Additionally, MEcPP stimulates the expression of IRE1a and bZIP60, contributing to UPRs via the CaM-binding transcription activator CAMTA3 (Figure 5). CUE1 facilitates the import of extra-plastid phosphoenolpyruvate (PEP) into chloroplasts, where it participates in tocopherol (vitamin E) synthesis from the aromatic amino acid tyrosine in *B. rapa* [29,108,109]. Tocopherols play a role in stabilizing primary miRNAs (like miR398), inhibiting nuclear exoribonucleases, and promoting acquired tolerance in plants (Figure 5). Overall, these findings underscore the critical role of retrograde signaling from chloroplasts in the molecular response to HS.

### 4.7. Molecular Mechanism and Genetic Control of HSRs in Crops

Crop productivity is declining because of global warming. Future agricultural growth depends on our ability to comprehend how crops respond to HS. The members of the Brassicaceae family are cultivated throughout the globe, in both temperate and tropical climates. Geographical adaptations and the acquisition of natural alleles that give resistance in various environmental niches have been experienced by *Brassica* species. The network of HS responses in *Brassica* species is described in this section.

### 4.8. Ca^2+^ Elevation Initiates Heat Signal Transduction

**Figure 6:**
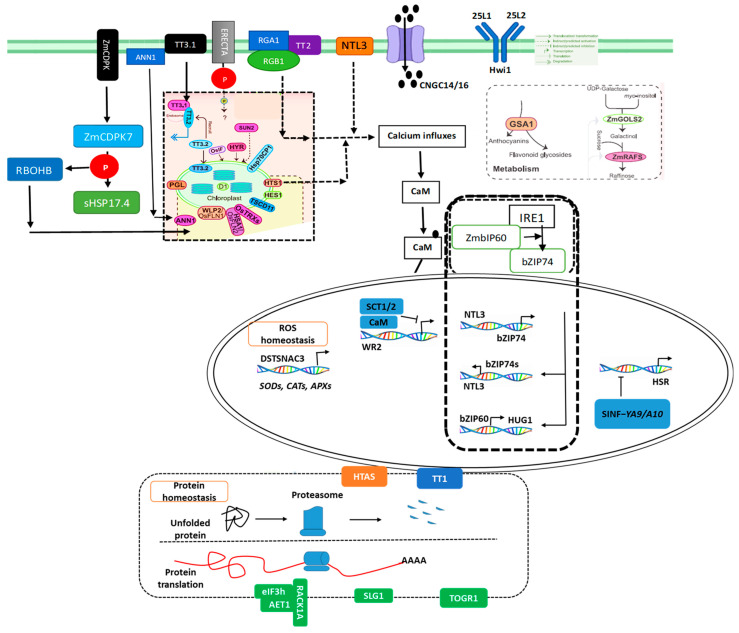
Crop molecular reactions to HS. CNGC14/16 promotes heat-induced Ca^2+^ influx in plants. The heat-induced rise in cytosolic Ca^2+^ content, which is then decoded by CaM-SCT1/2 interactions and inhibits WR2 transcription in plants, is another function of TT2. Upstream of the heat responses, ERECTA and Hwi1 operate as putative receptor-like kinases. In reaction to HS, TT3.1 moves from the plasma membrane into the endosomes, where it ubiquitinates and recruits TT3.2, blocking the formation of mature TT3.2 in plant chloroplasts. Heat-induced membrane-to-nucleus translocation is carried out by NTL3, which also transcriptionally activates bZIP74 and other downstream genes. In plants, the PSII component D1 is sensitive to HS, as are IF, PGL, and HES1, which are necessary for maintaining the chloroplast ultrastructure. Similar heat tolerance mechanism was observed in grain crops [5].

In response to HS, plants alter their membrane state, activate receptors, raise cytosolic Ca^2+^ levels, and move proteins, among other things. Heat-induced Ca^2+^ influx in *B. napus* has been shown to need cyclic nucleotide-gated ion channels CNGC14 and CNGC16 (Figure 6). Comprehensive analyses of the annexin (ANN) gene family in *B. rapa*, *B. oleracea*, and *B. napus* reveal their roles in stress response [110]. According to earlier studies, G-proteins are crucial for the development and production of rapeseed, but it is yet unknown how relevant they are for thermo-tolerance [111]. The TT2 (GS3) gene encodes a gamma subunit of the G protein. Normal TT2 function and cytosolic Ca^2+^ levels are associated with the SCT1-CaM interaction. Increased Ca^2+^ levels encourage CaM to bind to SCT1 (sensing Ca^2+^ transcription factor 1), which inhibits the target gene’s activity (BnWR2, wax synthesis regulatory 2), resulting in thermo-sensitivity and wax decrease under HS (Figure 6). By lowering heat-induced Ca^2+^ levels, the TT2HPS32 null allele breaks the CaM-SCT1 bond and lifts *B. rapa* SCT1 repression in broccoli [112]. Since TT2 is a special negative regulator of thermo-tolerance, it may have a significant impact on agricultural productivity.

### 4.9. Plasma Membrane-Localized Proteins Translocate into Other Cell Components to Transduce Heat Signals

Communication between organelles and the detection of extracellular heat signals at the plasma membrane are crucial for plant stress responses. For instance, in response to heat treatment, *B. rapa*, *B. oleracea*, and *B. napus* show cytosolic accumulation of ANN1 from the cell periphery [110,113], highlighting its role in the stress response (Figure 6). Additionally, during HS, transcriptome data revealed that the transcription factor NTL3 migrates from the plasma membrane to the nucleus, activating transcriptional responses in rapeseed [114]. In Chinese cabbage, the Ca^2+^-dependent protein kinase CDPK7 translocate from the plasma membrane to the cytoplasm during HS. It phosphorylates RBOHB (Respiratory Burst Oxidase Homolog B) and small heat shock protein sHSP17.4, thereby enhancing their expression levels. The expression of these proteins is regulated by both abscisic acid and heat, with RBOHB also serving as a feedback regulator upstream of sHSP17.3 and CDPK7 [115].

### 4.10. HSF–HSP-Mediated Transcription Activation in the Nucleus

HS transcription factors, specifically HSPs and HSFs, exhibit multiple isoforms that form complexes to regulate downstream transcription levels. In crop plants, a systematic HSF–HSP network has been well-characterized (Figure 6). In Chinese cabbage, a genome-wide analysis revealed that Hsf proteins such as HsfA1a, HsfB1, HsfA7s, and HsfA6b can bind to the MIR169 promoter, leading to increased levels of miR169s [34]. The rise in miR169 levels subsequently reduces NF-YA9/A10 levels, alleviating their suppression on HSR genes like HsfA2, HsfA3, and HsfA7 [116,117]. Additionally, the protein HAC1, similar to the CREB-binding protein, forms a ternary complex with HSFs, facilitating HSR activation and maintenance [118]. In rapeseed, HSP1 enhances thermo-tolerance, and a positive feedback loop involving HSP101 and HSA32 (HS-associated 32-kD protein) supports long-term acquired thermo-tolerance [119]. Similarly, *ZmHSFA2* undergoes post-translational modifications (PTMs) in response to heat, forming active homo-trimers in maize [120]. To attenuate HSRs and establish negative feedback regulation, *ZmHSFA2* promotes the production of HSBP2 (HEAT SHOCK BINDING PROTEIN 2), which interacts with the trimers to disrupt their binding to the heat shock element, thereby modulating HSR activation.

### 4.11. Regulation of Protein Degradation and Translation Maintain Protein Homeostasis Under HS

A surplus of cytotoxic proteins and abnormal protein translation can disrupt protein homeostasis, leading to cell damage and potentially cell death. To mitigate these effects, plants must efficiently eliminate unfolded proteins [121]. One of the genes to be first found to be associated with crop thermo-tolerance is TT1, which encodes the a2 subunit of the 26S proteasome, a multi-subunit protease complex that selectively degrades ubi-quitinated proteins (Figure 6). The TT1CG14 allele reduces heat response fluctuations by buffering proteins in *B. rapa* and enhances the degradation of denatured proteins [122]. Transgenic plants overexpressing TT1CG14 from various species, including tall fescue Chinese cabbage and *Arabidopsis*, exhibit increased thermo-tolerance [34,123]. Similarly, in wheat, both HTAS and TT1 similarly function by effectively eliminating toxic proteins through the ubi-quitination pathway [124]. Meanwhile, in tomatoes, the protein SlCHIP (carboxyl terminus of the hsc70-interacting protein) targets misfolded proteins and encodes a chaperone-dependent Ub-E3 ligase, which positively regulates thermo-tolerance [125]. Maintaining protein homeostasis is also critical for plants exposed to high salt levels, necessitating normal protein translation. The stability of RNA is vital for effective translation (Figure 6), and the heat-induced DEAD-box RNA helicase TOGR1 enhances thermo-tolerance when overexpressed in transgenic Chinese cabbage [126].

### 4.12. Post-Translational-Modification

The shift in protein levels reflects how plants respond to HS. The SlSIZ1, a prominent SUMO E3 ligase, enhances thermo-tolerance by mediating the SUMOylation of HsfA1 in plants [127]. This post-translational modification affects HSF activity, crucial for managing HS responses effectively. Additionally, SlMPK1 (mitogen-activated protein kinase 1) contributes by phosphorylating SlSPRH1 (serine-proline-rich protein homolog). This phosphorylation decreases antioxidant defense protein levels, which can negatively affect tomato thermo-tolerance in *Arabidopsis* [128]. Moreover, receptor-related proteins such as Hwi1 and ERECTA translocate to the plasma membrane, connecting external heat signals to internal physiological responses, likely via phosphorylation mechanisms (Figure 6). This intricate interplay between signaling pathways and protein modifications underscores the complex regulatory networks that enable plants to adapt to HS effectively.

## 5. Maintaining Crop Productivity via Source-Sink Strategies

### 5.1. Source Strategies

#### Protecting Photosynthesis

**Figure 7:**
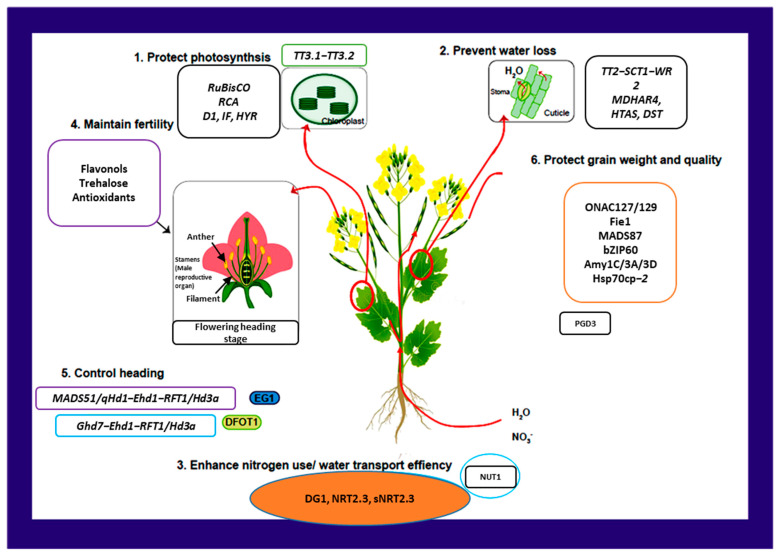
To enhance rapeseed production under HS, several strategies are proposed from both source and sink perspectives. The genetic modulation of photosynthetic components such as RuBisCO, RCA, D1, IF, HYR, and TT3.1–TT3.2 aims to improve heat tolerance in photosynthesis. Water retention can be increased by enhancing stomatal closure and cuticle deposition through pathways like MDHAR4, HTAS, DST, and TT2-SCT1-WR2. Additionally, improving nitrogen utilization, ABA transport, and water absorption efficiency via the NRT2.3, DG1, and NUT1 homologs can enhance survival rates. To support fertilization, the exogenous application of carbon and nitrogen metabolites, or boosting of endogenous levels, helps mitigate ROS accumulation in flowering organs. Optimizing the heading and flowering stages through pathways such as MADS51/qHd1–Ehd1–RFT1/Hd3a and Ghd7–Ehd1–RFT1/Hd3a, or genes like EG1 and DFOT1, can reduce heat damage. Finally, maintaining endosperm development and improving starch biosynthesis while inhibiting amylase activity can enhance grain weight and quality, regulated by genes like ONAC127/129, Fie1, MADS87, MADS57-Wx, DOF17–WCR1–MT2b, bZIP60, bZIP58–Amy1C/3A/3D, TT1, Hsp70cp-2, and PGD3 homologs. Collectively, these strategies aim to bolster the resilience and yield of rapeseed amidst rising temperatures in, as previous studies focusing on grain crops have indicated [5].

Although photosynthesis is crucial for biomass generation and plant growth, it is also extremely heat-sensitive. Under high temperatures, RuBisCO activity is greatly decreased by heat unstable RuBisCO activase (RCA). While the short isoform RCAS sustains RuBisCO initial activity under typical conditions, the long isoform RCAL contributes to photosynthetic adaptation under moderate HS [129]. Without lowering RuBisCO levels, rapeseed resistance to HS may be increased by increasing its RCA concentration (Figure 7). A key part of PSII, heat-inducible D1 is artificially driven by the *AtHSFA2* promoter to shield the chloroplasts of transgenic plants from HS [130]. By raising net CO_2_ assimilation rates, sufficient D1 supplementation can enhance biomass and grain yield [131]. By improving photosynthetic acclimation, the TT3.1-TT3.2 genetic module provides novel methods for creating heat-tolerant crops (Figure 7). In drought and high-temperature conditions, HYR increases grain output by activating genes involved in photosynthesis and altering carbon metabolism (Figure 7).

### 5.2. Sink Strategies: Guaranteeing Normal Fertility and Heading Stage

Addressing low pollen viability, abnormal fertilization, and insufficient grain filling should be prioritized in agricultural research, rather than focusing solely on vegetative growth declines. Fertilization under HS is influenced by two primary factors as follows: the timing of flowering or heading and the accumulation of toxic compounds in floral structures. Heat-induced ROS levels can lead to a 60% reduction in pollen germination, attributable to morphological and anatomical changes such as tapetum degradation, anther indehiscence, and pollen abnormalities in rice [132]. During HS, nitrogen and carbon metabolites play a crucial role in maintaining ROS homeostasis and facilitating successful fertilization in rice [133]. This application enhances the antioxidant system and sustains elevated levels of endogenous spermidine and spermine by reducing mRNA levels (Figure 7). Additionally, flavonols act as antioxidants and ROS scavengers, preserving redox balance and ensuring the integrity and growth of pollen tubes during HS (Figure 7). Proline and γ-aminobutyric acid, which modulate Ca^2+^-permeable membrane channels contribute to ROS scavenging, alongside flavonoids. Moreover, metabolites such as trehalose, sorbitol, glycine betaine, and polyamines indirectly reduce ROS levels by stabilizing antioxidant enzymes (Figure 7). The timing of blooming and heading is critical for effective fertilization. Genetically manipulating the timing of these processes by either advancing or delaying them can help plants mitigate the detrimental effects of high temperatures, thereby fostering heat adaptation in Chinese cabbage [134]. Several genes have been identified as regulators of heading, including Hd1, Ehd1, Ghd7, and Hd3a/RFT1. Collectively, these genes function as key regulators, forming the Ghd7–Ehd1–Hd3a/RFT1 pathway, which is vital for plant development [135].

Under high temperatures, the regulation of downstream RFT1 by Ghd7 diminishes due to decreased Ghd7 mRNA levels, facilitating the appropriate floral induction in rapeseed (Figure 7). Lipase EG1 (EXTRA GLUME 1) enhances floral phenotype resilience and acts as a buffer against heat-induced disruptions [136]. Consequently, *B. napus* maintains the normal expression of floral identity genes such as MADS1, MADS6, and G1 under HS conditions. The lodicule-localized DFOT1 (diurnal flower opening time 1) regulates the swelling of lodicules in coordination with several essential floral genes. DFOT1 interacts with pectin methyl esterase (PME) to enhance its activity, thereby optimizing the timing of blooming (Figure 7). By integrating genetic approaches to control blooming, the timing of crop phase transitions can be improved, ultimately enhancing reproductive success.

## 6. Maintaining Grain Yield

HS often leads to grain-filling impairment, resulting in reduced grain weight and quality. Therefore, a continuous nutrient flow from source to sink, as well as proper endosperm development, are critical for grain production under HS conditions. Sugar transport during HS is associated with a NAC-type transcription factor (Figure 7). The Polycomb Repressive Complex 2 (PRC2) is essential for endosperm development in cereals, with the transcriptional level of Fie1 encoding a component of the FERTILIZATION INDEPENDENT SEED PRC2 complex, which plays a pivotal role in determining grain width. The FERTILIZATION INDEPENDENT SEED–PRC2 complex negatively regulates MADS78/79 transcription factors, which delay endosperm cellular development in *Solanum lycopersicum* [137]. Additionally, MADS87 negatively influences thermo-tolerance while positively affecting seed size (Figure 7). These genes present valuable genetic resources for developing thermo-tolerant crops, warranting exploration for potential allelic variations.

The endosperm is crucial for determining both the weight and quality of grain seeds. It primarily consists of amylopectin (70–90%) and amylose (the remaining fraction), with amylose content serving as a key indicator of grain quality. High temperatures can adversely affect amylose levels, but maintaining consistent amylose contents during HS can enhance grain quality by promoting the efficient pre-mRNA splicing of the Wx (waxy) genes (Figure 7). The Wx gene is a significant regulator of amylose content. While MADS57 promotes spikelet fertility during the early filling stage, it negatively affects amylose concentration in the endosperm during HS. This dichotomy underscores the importance of potentially reducing MADS57 levels in the endosperm to enhance grain quality under HS (Figure 7). Under HS, plants exhibit enlarged protein storage vacuoles but lack sufficient storage proteins. Abnormal amyloplast development and increased amylase expression levels contribute to air hole formation in dehydrating kernels, leading to chalkiness, a negative trait in grain quality [138]. Hsp70cp-2 facilitates protein import into the amyloplast; however, mutations in the Hsp70cp-2 gene only improve grain quality under HS (Figure 7). Chalky grains arise from excessive alpha-amylase production in non-HS-exposed plants. Temperature correlates positively with alpha-amylase expression and enzymatic activity (Figure 7). Upstream, bZIP58 inhibits alpha-amylase activity while promoting seed protein storage and starch synthesis. The heat-induced alternative splicing of bZIP58 results in truncated proteins with reduced transcriptional activity compared to the full-length bZIP58a protein, contributing to chalkiness (Figure 7). Oxidative damage can also lead to chalkiness; the metallothionein gene MT2b interacts with and stabilizes the F-box protein encoded by WCR1 (white-core rate 1), reducing reactive oxygen species accumulation. Allelic variations in the WCR1 promoter that enhance DOF17 binding activity have been shown to improve grain quality and decrease chalkiness (Figure 7). Additionally, heat-sensitive 6-phosphogluconate dehydrogenase PGD3 facilitates starch accumulation in maize, exhibiting increased activity and heat resistance when correctly targeted to the appropriate subcellular compartment, thereby mitigating crop failure due to HS in rapeseed [139]. The orthologs of these genes in *Brassica* species may provide further avenues for research [140]. In summary, elucidating these molecular pathways enhances our understanding of grain seed development in environments with fluctuating temperatures.

## 7. Concluding Remarks and Future Perspectives

Despite advances in understanding the effects of various biotic and abiotic stress factors on different members of the Brassicaceae family, significant knowledge gaps remain regarding the impact of HS on *Brassica* species during its yield-determining reproductive stages, where more in-depth analysis is urgently needed. The differential expression trends of the key genetic and epigenetic components observed across species and within cultivars under different abiotic stresses highlight the need for further investigation. Additionally, variations in the intensity, timing, and duration of HS have been shown to elicit distinct plant responses. To fully understand its effects on *B. napus*, regional studies on local cultivars are essential to replicate field-specific conditions and assess the crops’ response in particular geographical areas. The mechanisms underlying plant responses to rising temperatures have been the subject of extensive research; however, a comprehensive understanding of these processes remains elusive. The way in which heat is perceived and subsequently translated into physiological and molecular responses is still not fully understood. In this study, we categorize thermo-sensing and thermal responses into distinct sections to better elucidate these interconnected phenomena and enhance our grasp of the underlying biological mechanisms. While thermal response processes are often more complex and contentious, it is essential to acknowledge the cooperative nature of thermal sensing and responses.

Membrane proteins, critical for thermo-sensing, are characterized by the presence of both intracellular kinase domains and extracellular ligand-binding domains, along with their association with rapid Ca^2^⁺ activation. However, due to the penetrative nature of temperature and the propagation of thermal waves, the definition of thermo-sensors may extend beyond the plasma membrane. Structural changes at the protein and RNA levels induced by temperature appear to be particularly pronounced when sensing direct increases in temperature. Thermo-morphogenesis, which occurs during slight temperature elevations, is closely associated with many identified thermo-sensors. Further investigation is necessary to determine whether similar mechanisms underpin HS perception. Another avenue for heat signal transduction involves subcellular translocation, particularly from the membrane to the nucleus, though the fundamental mechanisms facilitating this process remain largely unexplored. The proposed factors contributing to trans-organelle translocation, which characterizes thermo-sensors, include splicing, chaperone interactions, and lipid anchoring. These findings underscore the need for additional research to clarify the mechanisms by which plants detect temperature variations. We also propose various strategies to ensure or enhance crop tolerance to heat. Crop breeders require a comprehensive understanding of the intricate network of heat response mechanisms to address the global food security challenges posed by climate change. Future research should encompass the duration and intensity of temperature increases, variations in tissue temperatures, and the processes involved in heat shock recovery. Incorporating the concept of multifactorial stress combinations into future studies is advisable, as environmental stressors typically manifest as a combination of two or more abiotic and/or biotic factors. For instance, HS often co-occurs with drought stress, and insect larvae have adapted to produce additional cuticle proteins in response to global warming for effective heat acclimation. High temperatures can also compromise plant defenses. Consequently, future breeding initiatives should not solely focus on specific environmental stressors. Instead, there is a need to identify moonlighting regulators that concurrently manage disease resistance, insect tolerance, drought resilience, and thermo-tolerance. This approach is essential for overcoming the post-Green Revolution agricultural challenges and discovering innovative methods to enhance sustainable crop production and acclimation in the face of climate change.

## Data Availability

Not Applicable.

## Abbreviations

ABA	Abscisic acid
ANNs	Annexins
ATAD3	ATPase family AAA domain-containing protein 3
ATI3	ATG8-INTERACTING PROTEIN3
CaM	Calmodulin
cAMP	Cyclase produces cyclic AMP
CBK3	Calmodulin-binding protein kinase 3
cGMP	Cyclic guanosine monophosphate
CK1	(CASEIN KINASE 1)
CLC2	CLATHRIN LIGHT CHAIN 2
CMT2	Chromomethylase 2
CNGCs	Cyclic nucleotide-gated channels
CNGCs	Cyclic nucleotide-gated channels
CYP79B2	Cytochrome P450 79B2
DAG	Diacylglycerol
DFOT1	Diurnal flower opening time 1
DGK	Phospholipase C/DAG kinase
DREB2A	DEHYDRATION-RESPONSIVE ELEMENT BINDING 2A
EG1	EXTRA GLUME 1
ELF3	EARLY FLOWERING 3
ER	Endoplasmic reticulum
FT	Flowering locus T protein
GAPC	Glyceraldehyde-3-phosphate dehydrogenase
GRF7	GROWTH-REGULATING FACTOR 7
HDA6	Histone deacetylase 6
HIL1	Heat-inducible lipase 1
HS	Heat stress
HSA32	HS-associated 32-kD protein
HSBP2	HEAT SHOCK BINDING PROTEIN 2
HSFs	Heat shock factors
HSPs	Heat shock proteins
HSR	Heat shock response
JA	Jasmonates
JUB1	JUNGBRUNNEN 1
LLPS	Liquid–liquid phase separation of proteins.
lncRNAs	Long non-coding RNAs
LNG1	Longifolia 1
LNG2	Longifolia 2
MBF1c	MULTIPROTEIN BRIDGING FACTOR 1C
mRNAs	Messenger RNAs
MSH1	MutS homolog 1
NBR1	NEXT TO BRCA1 GENE 1
ncRNAs	Non-coding RNAs
NF-YC10	Nuclear factor Y subunit C10
NRD	Negative regulatory domain
Pas	Phosphatidic acids
PE	Phosphatidylethanolamine
PEP	Phosphoenolpyruvate
PhyB	Phytochrome B
PIF4	PHYTOCHROME-INTERACTING FACTOR 4
PIF7	PHYTOCHROME-INTERACTING FACTOR 7
PLC/DGK	Phospholipase C/diacylglycerol [DAG] kinase generate
PLC	Phospholipase C
PLD	Phospholipase D
PME	Pectin methylesterases
PRC2	Polycomb Repressive Complex 2
PROMPTs	Promoter upstream transcripts
PTMs	Post-translational modifications
RBGD2	RNA-binding glycine-rich D2
RBOHB	Respiratory burst oxidase homolog B
RBOHD	Respiratory burst oxidase homolog D
RCA	RuBisCO activase
RCD1	RADICAL-INDUCED CELL DEATH 1
RdDM	RNA-directed DNA methylation
RNA Pol II	RNA polymerase II
ROS	Reactive oxygen species
SA	Salicylic acid
SCT1	Sensing Ca^2+^ transcription factor 1
SGs	Stress granules
sHSPs	Small heat shock proteins
SlCHIP	Carboxyl terminus of the hsc70-interacting protein
SlMPK1	Mitogen-activated protein kinase 1
SlSPRH1	Serine-proline-rich protein homolog
SPL	SQUAMOSA PROMOTER BINDING PROTEIN-LIKE
TAA1	L-tryptophan--pyruvate aminotransfer-ase 1
TT	Thermo-tolerance
UPR	Unfolded protein responses
UPS	Pathways ubiquitin (Ub)-26S proteasome system
YUCCA8	Indole-3-pyruvate monooxygenase 8
